# Supplementary material for: The Novel Role of SOX2 as an Early Predictor of Cancer Risk in Patients with Laryngeal Precancerous Lesions
Source: Cancers (Basel). 2019 Mar 1;11(3):286. doi: 10.3390/cancers11030286 (PMC6468607; doi:10.3390/cancers11030286)
Supplement: Supplementary file 1 [file cancers-11-00286-s001.pdf]

Supplementary Materials: Novel Role of Sox2 as Early Predictor of Cancer Risk in Patients with Laryngeal Precancerous Lesions

Rocío Granda-Díaz, Sofía T. Menéndez, Daniel Pedregal Mallo, Francisco Hermida-Prado, René Rodríguez, Laura Suárez-Fernández, Aitana Vallina, Mario Sánchez-Canteli, Aida Rodríguez, M. Soledad Fernández-García, Juan P. Rodrigo and Juana M. García-Pedrero

A

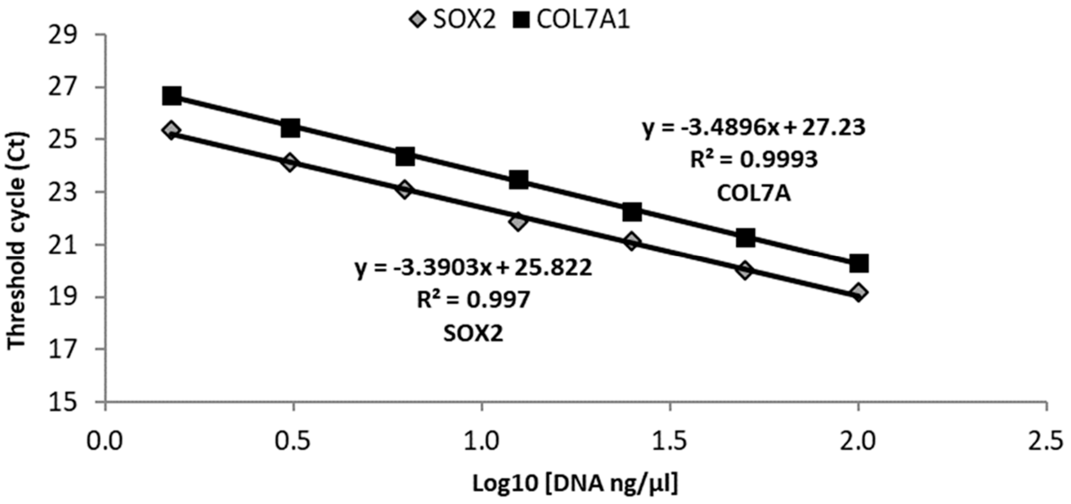

B

Gene	Slope	Efficiency
SOX2	-3.390	1.97
COL7A1	-3.489	1.93

Figure S1. Standard curve showing the Log10 DNA amount, in ng per µL, plotted against threshold cycle (Ct) for different dilutions of genomic DNA in PCR-grade water by real-time PCR (**A**). The table shows the efficiency of amplification for each primer pair, calculated using the slope of the corresponding standard curve (**B**).

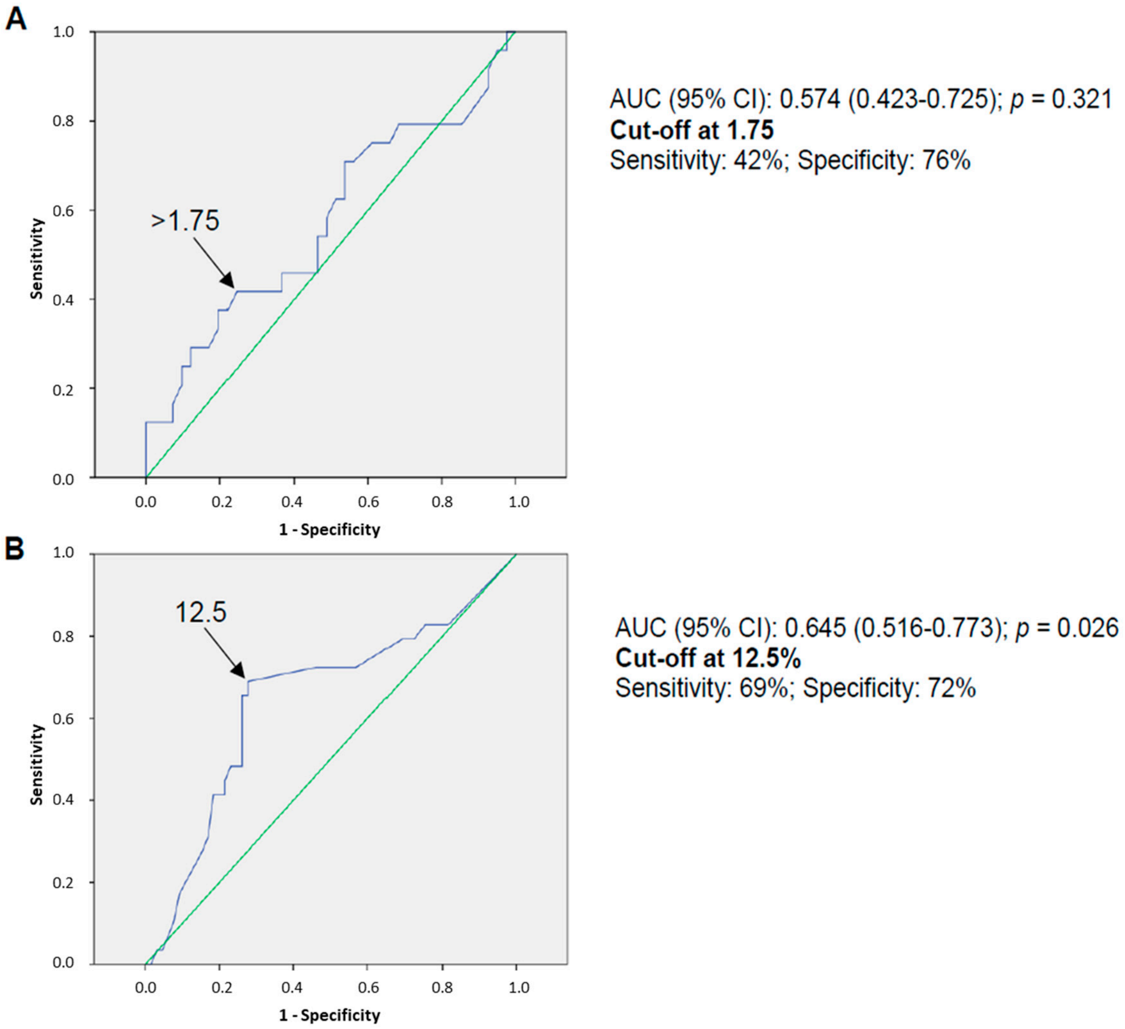

Figure S2. Determination of the optimal cut-off values for *SOX2* amplification and SOX2 expression by receiver operating characteristic (ROC) curve analysis. ROC curves for *SOX2* amplification (**A**) and SOX2 expression (**B**) (as percentage of positive nuclei). The optimal cut-off values calculated were 1.75 for *SOX2* amplification and 12.5% for SOX2 expression. The area under de curve (AUC), with 95%CI, the *p* values, and the sensitivity and specificity calculated for each optimal cut-off are shown.

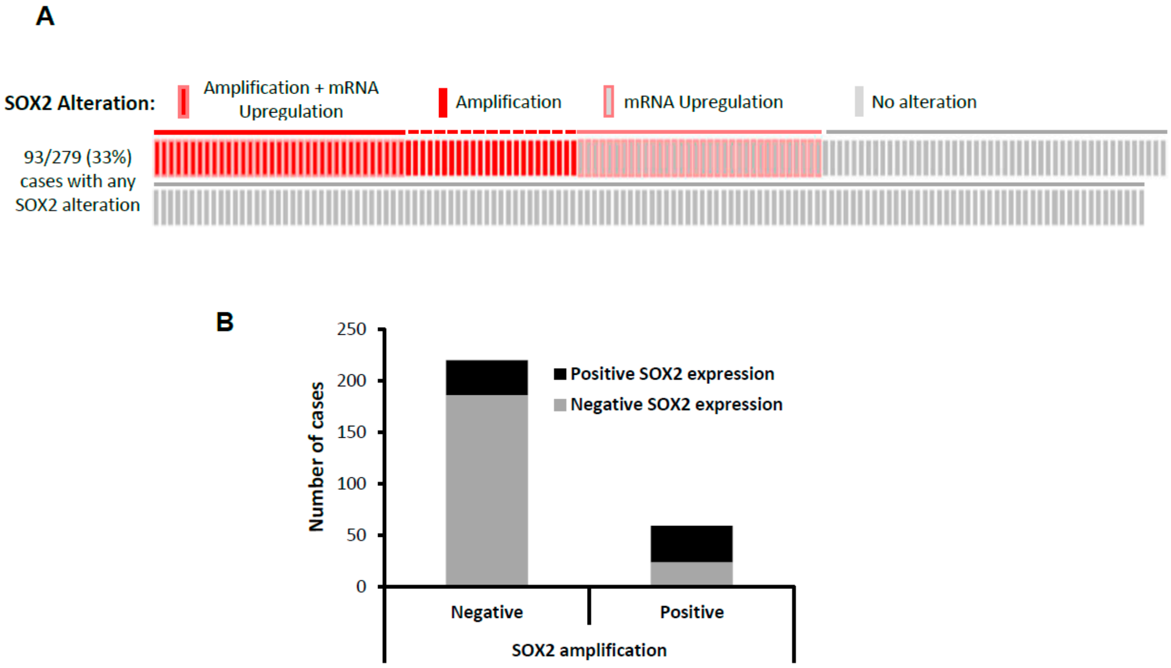

Figure S3. Analysis of *SOX2* gene amplification and mRNA expression from the TCGA cohort of 279 HNSCC patients [44] using cBioPortal [45]. Schematic representation (**A**) showing the number of cases with gene amplification and/or mRNA upregulation or no alteration. Correlations between SOX2 expression and gene amplification in this cohort (**B**).

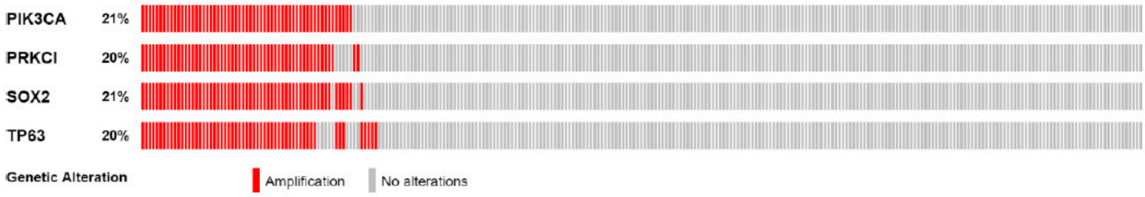

Figure S4. Gene amplification analysis of *SOX2* and other driver genes (*PIK3CA, PRKCI* and *TP63*) mapping at 3q26 amplicon using the TCGA cohort of 279 HNSCC patients [44] and the platform cBioPortal [45]. Oncoprint illustrates frequent co-amplification of all these genes.
